# Establishing a Community Air Monitoring Network in a Wildfire Smoke-Prone Rural Community: The Motivations, Experiences, Challenges, and Ideas of Clean Air Methow’s Clean Air Ambassadors

**DOI:** 10.3390/ijerph17228393

**Published:** 2020-11-13

**Authors:** Amanda Durkin, Rico Gonzalez, Tania Busch Isaksen, Elizabeth Walker, Nicole A. Errett

**Affiliations:** 1 Department of Environmental and Occupational Health Sciences, University of Washington, Seattle, WA 98195, USA; amddurkin@gmail.com (A.D.); gorico@uw.edu (R.G.); tania@uw.edu (T.B.I.); liz@mvcitizens.org (E.W.); 2 Clean Air Methow, Methow Valley Citizens Council, Twisp, WA 98856, USA

**Keywords:** wildfire smoke, air quality, clean air monitoring network, community science

## Abstract

In response to wildfire-related air quality issues as well as those associated with winter wood stove use and prescribed and agricultural burning, Clean Air Methow’s Clean Air Ambassador program established a community air monitoring network (CAMN) to provide geospatially specific air quality information and supplement data generated by the two Washington State Department of Ecology nephelometers situated in the area. Clean Air Ambassadors (CAAs) were purposefully selected to host low-cost air sensors based on their geographic location and interest in air quality. All 18 CAAs were interviewed to understand their motivations for participation, experiences using the data, challenges encountered, and recommendations for future project directions. Interview transcripts were coded, and a qualitative analysis approach was used to identify the key themes in each domain. The reported motivations for participation as a CAA included reducing personal exposure, protecting sensitive populations, interest in air quality or environmental science, and providing community benefits. CAAs used CAMN data to understand air quality conditions, minimize personal or familial exposure, and engage other community members in air quality discussions. Opportunities for future project directions included use for monitoring other seasonal air quality issues, informing or reducing other pollution-generating activities, school and community educational activities, opportunities for use by and engagement of different stakeholder groups, and mobile-friendly access to CAMN information. Limited challenges associated with participation were reported. Additional research is necessary to understand the community-level impacts of the CAMN. The findings may be informative for other rural wildfire smoke-prone communities establishing similar CAMNs.

## 1. Introduction

As climate change leads to the increased frequency, duration, and intensity of wildfires [1,2,3,4] and longer regional fire seasons [5,6], wildfire-associated air quality impacts are becoming an increasing public health concern for rural communities across the Western United States. Three recent review articles, documenting the human health impacts of wildfire smoke across over 60 epidemiologic studies, found wildfire smoke exposure to be associated with respiratory morbidity and all-cause mortality [7,8,9]. The cardiovascular health impacts of such events have also been documented; however, evidence pointing toward an association is mixed [8,10]. Children, the elderly, and those who spend large amounts of time outdoors are at highest risk of exposure [11,12].

Geospatially specific wildfire smoke exposure information is needed to better understand exposure–outcome relationships, as well as to inform protective actions and behaviors. Conventional air monitoring, which employs expensive, sparsely situated monitors to ensure compliance with federal or state regulations, has limited ability to detect air quality “hot spots” or monitor the spatial or temporal air quality impacts of extreme events, especially those excluded from air quality regulation, such as wildfires [13,14]. Furthermore, given that the purpose of conventional monitors is to detect the ambient air quality levels for regulatory purposes, they may not be situated in rural areas subject to wildfire smoke impacts [9].

The availability of low-cost, portable air sensors has the potential to provide real-time information about air quality with a geospatial resolution that can improve wildfire smoke exposure models [9]. Moreover, they can provide individuals and communities with the data to understand their environmental exposures and help them develop individual and community-based strategies to reduce risk [15].

Communities across the globe have created networks of low-cost air sensors to supplement conventional monitors [13,14,16]. In fact, California’s Assembly Bill 617 established a “community-focused action framework to improve air quality in communities most impacted by air pollution” that includes community air monitoring [17].

A community air monitoring network (CAMN) has been previously described “as a collection of air monitors located throughout a community or region that: is established and operated by a community-based organization, is intended to be long-term; aims to measure ambient air quality (versus measuring pollutants from a single specific source); continuously measures air pollution” and “collects data that are made available in real-time to the public” [18]. The establishment and use of low-cost CAMNs provide a unique opportunity to engage community members in data collection, analysis, and interpretation to increase information about the air quality impacts of wildfire smoke, a form of citizen science [19]. In the northwest, community and governmental organizations in rural communities responsible for or interested in the public health impacts of wildfire smoke have become increasingly interested in using CAMNs to obtain more geospatially specific information about wildlife smoke exposure. Given the sparse situation of state and federal monitors, these networks have the potential to identify “safer” air spaces within a monitor’s service area. To date, however, organizations interested in establishing these networks in response to wildfire smoke concerns have received relatively little guidance regarding network establishment, including around the recruitment and retention of sensor hosts.

Engagement in citizen science projects has been described to influence community health through a variety of mechanisms, including impacts on health literacy, empowerment, community building, social capital, attitudes, norms, and values [20]. Specific to the establishment of CAMNs, engaging community members in program design has been reported to have resulted in trust in and usability of sensor-generated data among community residents, while concurrently generating data can be used in scientific analyses [21]. Yet, to the best of our knowledge, there has not yet been empirical research on the motivations or impacts of those participating in a CAMN project as sensor hosts. This study sought to better understand the motivations of and impacts on individuals who volunteer to serve as sensor hosts in CAMN projects, especially those around wildfire smoke. Here, we present the results of a thematic qualitative analysis of interviews with volunteers who have situated low-cost sensors in their homes or workplaces as part of a wildfire smoke-related community air monitoring project in the Methow Valley, a rural community in northeastern Washington State, USA.

### Clean Air Methow’s Clean Air Ambassador Program Case Study

The Methow Valley community, a rural community located in Washington’s Okanogan County, has had longstanding issues with air quality associated with silviculture and agriculture burns in spring and fall and wood stove use in winter. In response to these concerns, Clean Air Methow (formerly the Methow Valley Clean Air Project) was established by the Methow Valley Citizens Council in 2013. The interest in and activities of the project have expanded because of the severe wildfire smoke impacts experienced in recent years. To illustrate, in August 2018 the Methow Valley experienced air quality categorized as “Unhealthy for Sensitive Groups” or worse on Washington’s Air Quality Advisory (WAQA) index for 21 consecutive days. Twisp, the home of 1153 of the Valley’s 5889 residents (based on American Community Survey 2014-2018 5-Year Estimates) [22,23], experienced air quality at “Unhealthy” or worse levels for all of August, with peaks as high as 300 μg/m³ for fine particulate matter (PM_2.5_). These levels well exceed what the WAQA considers “Healthy” air (categorized as 12 μg/m^3^ (PM_2.5_) or less). Previously, in August and September of 2017, the same pattern was observed, with over two weeks of the air quality reaching WAQA levels of “Unhealthy for Sensitive Groups” or worse [24].

**Figure 1:**
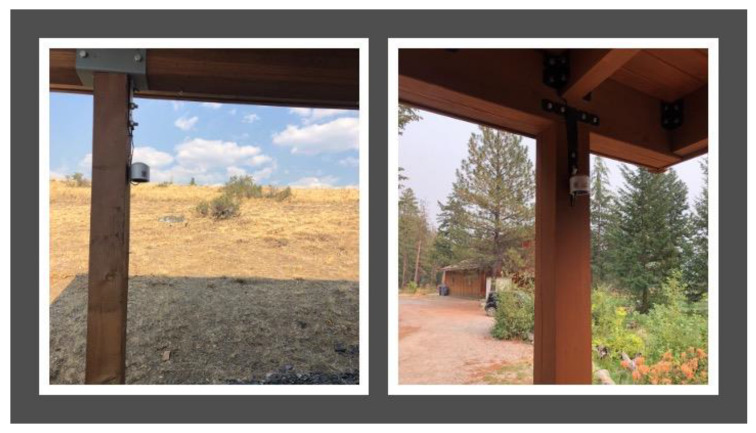
Examples of CAA (Clean Air Ambassadors) Purple Air monitors.

In response to community concerns about wildfire smoke air quality impacts, Clean Air Methow, in partnership with the University of Washington’s Department of Environmental and Occupational Health Sciences, established a CAMN of 20 Purple Air monitors in summer 2018. The program, known as the Clean Air Ambassador Program, sited 20 PurpleAir monitors (Figure 1) with volunteers across the valley to provide supplemental and geospatially specific air quality information to that provided by the two Washington Department of Ecology nephelometers situated in the region [25]. Real-time measurements from the Methow Valley’s CAMN and more information about Clean Air Methow can be found on the organization’s website: www.cleanairmethow.org.

At $279/unit (as of September 2020), PurpleAir monitors can provide geospatially specific air quality information at a relatively low cost [26]. The Purple Air monitor uses dual laser counters to provide reliable particulate readings. The PurpleAir unit includes built-in WiFi to automatically upload data for download and display on the PurpleAir map approximately every 80 seconds [26]. As such, data from the monitors are publicly available. Clean Air Methow’s website links to an interface hosted on the Purple Air Monitoring’s website that includes only the monitors in the valley.

As low-cost air monitors have been shown to overreport the PM_2.5_ concentration in lab settings (AQMD), a correction factor was first calculated by co-locating two Purple Air Monitor with two nephelometers that are part of the Washington Department of Ecology’s permanent, maintained ambient air monitoring network. After the data were collected, a linear regression analysis was conducted to estimate the correction factor between the sensor-generated and nephelometer-generated data.

The remaining 18 monitors were placed in homes, businesses, or places of employment (including schools) of “Clean Air Ambassadors (CAAs)” or citizen scientist volunteers who committed to maintain and promote information about the monitor and the CAMN. Clean Air Methow staff recruited CAAs because of their location in Methow Valley and their interest in monitoring air quality. In this rural community, some locations did not have access to WiFi, which limited PurpleAir monitor placement to areas with WiFi.

A member of the project team (AD) traveled to the homes of the CAAs to help install the monitors and answer any CAA questions. CAAs have since communicated with Clean Air Methow on an as-needed basis, largely related to maintenance and troubleshooting. As Clean Air Methow seeks to grow and sustain the CAA program, they hired a part-time staff member in the spring of 2020 to increase the quantity and quality of engagement with CAAs.

## 2. Materials and Methods

Eighteen CAAs (100%) were interviewed from 16 August 2018, to 30 October 2018. Contact information was provided by Clean Air Methow. Interviews were conducted by a University of Washington Environmental Health undergraduate student serving as a summer research intern at Clean Air Methow (AD), who was co-supervised by University of Washington faculty members with expertise in exposure science (AD) and qualitative methods (NE) and Clean Air Methow staff (EW).

An interview guide was developed in advance and used to structure conversations. Questions were designed to elicit discussion about volunteer motivations to participate as a CAA with Clean Air Methow, the ways in which CAAs used the information from the low-cost sensors, the CAA’s ideas about improving the project or using the sensor information in the future, and how the CAA’s participation in the program influenced their ability to make decisions or take actions to protect their health or that of their family during wildfire smoke events. Interviews were conducted in person (*n* = 16) or over the phone (*n* = 2) and lasted approximately 20 minutes each. The following questions were asked during all interviews: What motivated you to become a Clean Air Ambassador? Is air quality something that impacts your life on a daily basis? If so, how? Is any member of your family in a sensitive population? If you have accessed the network, how have you been using the information so far? Has it influenced any of your decisions? What do you plan on doing with the information from the sensor in the future? Do you have any other ideas about the project (from friends?) Are there any barriers or challenges so far with the air monitoring project? If so, how can we help?

Interviews were recorded using a handheld recording device and transcribed. An initial codebook was developed based on the interview guide and citizen science literature. Additional codes were added following familiarization with the transcribed text. The transcribed interview text was coded using a consensus-based approach, with all coding decisions made collaboratively by 2–3 coders (AD, RG, NE), using NVivo qualitative data analysis software from QSR International Pty Ltd. Version 11, 2015 (Burlington, MA, USA). The coded text was thematically analyzed (AD, RG) to identify the themes and trends within and across codes. A single member of the author team developed summaries of key themes by code (AD or RG). Two members of the author team reviewed and amended the summaries to ensure they comprehensively presented key themes, as well as the discussion of minor themes and counterpoints, and included relevant quotations that demonstrated key points (AD or RG, and NE).

Interviewees provided verbal consent prior to participating in this study. The University of Washington Institutional Review Board approved this study, which qualified for expedited review (“minimal risk”, Category 5).

## 3. Results

The majority of participants that hosted a sensor at their place of residence or private business (*n* = 17) had two people in their household (59%), had no children under 18 in the household (65%), were married (71%), were white (88%), were female (71%), were over 40 years of age (82%), had at least a bachelor’s degree (83%), or were employed full or part time (64%) (Table 1). The CAAs reported a variety of motivations for participation in the Clean Air Methow Clean Air Ambassador Program. They described several ways that they use the data generated by the CAMN and identified the impacts of their participation. They recommended next steps and future directions for the project and reported minimal barriers, challenges, or concerns about the CAMN (Table 2).

### 3.1. Motivations for Participation

The participants described pervasive impacts of wildfire smoke exposure on health and daily activities. Many CAAs reported spending time outdoors as part of their livelihood, for exercise, or for enjoyment of nature. They described motivations for participation as a CAA stemming from interests in reducing personal exposure and using the information to make decisions to avoid exposure.

The participants described perceived personal and familial susceptibility to wildfire smoke as a motivator for participation. One participant stated, “Well it’s all because of [child’s] asthma and we track it a lot, we’ve tracked it for years.” Another stated, “Knowing that every summer there’s going to be huge impacts in the air quality most likely and we want to know immediately if we need to bring the children inside. Mostly it’s just keeping the children safe.” More than a third of CAAs reported having children in their household, and two respondents reported that these children have pre-existing respiratory conditions that make them more sensitive to smoke. Several participants cited their own pre-existing health conditions or sensitivities to wildfire smoke. Some CAAs mentioned fatigue, headaches, and difficulty breathing as a direct result of exposure to the smoke.

About half of the CAAs reported that they were motivated to participate because they wanted to know more about smoke conditions, local air quality, and/or how actual air quality correlated with the perceived air quality conditions. One CAA stated, “Well, I’m a numbers person and I’m also interested in my environment. So, I wanted to be able to monitor it here”.

Several CAAs expressed motivation stemming from a desire to benefit the community they live in, such as sharing information with the community, expanding the utilization of the community network, and planning school activities. One participant stated, “We have the wellbeing of our students in our hands and want to make sure we have good information to make decisions about when they should be outside and participating in sports or other school activities”.

### 3.2. Data Uses

Most participants said they used data to understand air quality conditions during smoke events, and several reported checking air quality information several times a day. CAAs also reported using the CAMN to avoid smoke exposure, either to protect themselves or at-risk populations in their households (e.g., children), to determine when to wear an N95 mask for protection from poor air quality, to find clean air in the valley, and to decide whether or not to exercise. CAAs also reported using information from the CAMN when deciding whether or not to go outdoors during wildfire smoke events. As one CAA stated, “I basically checked it every half hour if not more when it was really smoky just to know if we could go outside or not with the children.” Some individuals were able to decide to spend time in the valley or evacuate to another location based on the information from the CAMN. One respondent stated, “While we are away we check the monitor many times a day. We want to know when we will be able to return to our home”.

A few CAAs reported that, despite knowing about the poor air quality, they or others did not have the option to avoid it because of the tasks they needed to complete or work outdoors or because of a lack of resources. One CAA described her own need to work outdoors despite the air quality, “I mean basically because this is our livelihood so we don’t really have a choice but you know wearing mask, um, once we got into that second week.” Another stated, “... I can’t just hole up inside I’ve got irrigation to change and animals to take care of so I have to be outside, so yeah, it definitely impacts me. I have been wearing a mask. ”Another acknowledged the challenges others face: “I feel for folks who have to open their windows or can’t afford an air purifier or whatever else it is they’re living in it day to day and do they know”.

Half of the CAAs mentioned that they had shared the data, including with the community or with persons visiting the Methow Valley, and that the CAMN had sparked community conversations and discussion around air quality and exposure reduction. One CAA stated, “It comes up in conversation a lot. We’re proud we have a little monitor.” Interestingly, one participant described how topics of conversation about the CAMN included identifying opportunities to engage those without knowledge about smoke risk or resources to limit exposure.

“I know I talked with some other friends, you know there’s those of us who have the resources to make different decisions but how do we help those who maybe don’t and don’t understand that this is a real health threat, so I think there’s some of that being tossed around, which I think the whole project has helped initiate those conversations just because somebody is paying attention to them”.

### 3.3. Future Project Ideas

The CAAs reported interest in expanding the use of the CAMN to understand winter air quality issues, including related to inversions and outdoor burning, and to explore trends in air quality over time. The participants described the network’s influence on their awareness of other sources of pollution, and/or the potential for the network to inform individual and community-level awareness and decisions about pollution-generating and reducing activities and investments. One participant suggested that the Purple Air website be linked to from other community websites to increase awareness, including about other air quality impacts such as woodstove use. One participant stated.

“Now we’ll have this and if people are burning a lot we’ll know that and it can help people make decisions about whether or not they want to burn, whether or not they want to invest, if the county wants to invest, in a composting facility so people don’t have to burn or some larger scale chipping operation. There’s that and there’s also Forest Service is probably going to want to do more and more prescribed burns to reduce the fuel load in the forest, and it will help them decide when is the [air] contamination too great in the valley”.

The participants highlighted different opportunities for stakeholder groups to use the information generated by the monitors, including local businesses highlighting the availability of clean air in the valley and sports coaches making decisions about whether to practice indoors or outdoors or to cancel practice. CAAs indicated that they were interested in the use of the CAMN to increase the community’s awareness and knowledge about air quality issues and in community educational events about air quality.

The participants described the potential value of the CAMN for educational purposes—for example, by using the data in school to explain the air quality impacts of wildfire smoke. One respondent explained the educational benefits of the network: “A student seeing a number and knowing that’s [their] school can make it more real. It becomes very personal at that point. To be able to physically look at it [the smoke] and then to be able to talk about that one on one with a teacher and really understand it.” The potential secondary impacts of using school-based education on parents and families were also acknowledged, “Well I think because of where these monitors are I feel like it would be a great education tool to bring into the schools and to begin with kids because they will take it home to their parents whether the parents believe it or not.” One discussed the curriculum opportunities for comparative data analysis from monitor-generated data in different geographic areas.

CAAs suggested increasing the number of low-cost sensors as part of their own CAMN and expressed an interest in the project spreading to more areas within the state and country. A few participants cited specific locations where they felt additional sensors would be useful, including outside of the community to inform decisions about whether or not there is clean air in other locations. In addition to information provided by the CAMN about outdoor air, about a third of the CAAs were also interested in personal or project-coordinated indoor air monitoring in the future.

The majority of the CAAs were interested in the development of an app or “mobile friendly experience” to check the air quality information generated by the CAMN. They reported interest in additional information about the PurpleAir monitor’s “correction factor” with the state-situated nephelometers in the region. There were some people who wanted to see improved PurpleAir website clarity and for the data presented on the website to reflect an applied correction factor.

### 3.4. Project Challenges

Less than half of the CAAs reported barriers or challenges with using the CAMN or participating as a CAA. Commonly reported barriers included challenges using or confusion with the map interface display. One participant also noted that accessibility was limited to people with internet access and that signage was likely still important, although difficult to maintain. A few CAAs also reported concerns about lack of data if the monitor lost electricity or WiFi access. One CAA wished that the PurpleAir monitor could be hardwired via an ethernet cord versus reliant on WiFi. Another suggested that some potential CAAs might not like the aesthetics of the monitors, saying, “I suppose some people won’t like having it aesthetically, but you’ll find people who do”.

One participant described interactions in the community, where she was told the monitors were inaccurate, and wanted more information so that she could educate the community. She stated, “I would like it more described to me so that I can be an educator to those in the community as an ambassador. Because I want the most accurate information but I also want to understand it so I can teach that back…”.

## 4. Discussion

This innovative exploration of the motivations and experiences of community members volunteering to serve as CAAs for Clean Air Methow’s CAMN project can inform other rural communities in the establishment of similar CAMN projects. Clean Air Methow CAAs were motivated to participate as citizen science volunteers for a variety of reasons, including opportunities to reduce personal exposure, protect sensitive populations, learn more about air quality, and participate in a project with community benefits. The CAAs reported using the data to understand air quality conditions, determine personal protective actions, engage fellow community members, and promote air quality awareness. They made several recommendations to expand and enhance the CAMN and cited few barriers to their participation or challenges associated with the implementation or use of data generated from the CAMN.

CAAs reported using the CAMN to avoid poor air quality and to inform protective actions. However, there is limited evidence about the protective value of interventions that promote incremental breaks from air pollution exposure (e.g., the temporary use of N95 masks or the use of clean air shelters) in the context of high baseline levels of exposure and in preventing long-term health outcomes. Moreover, there is limited evidence about how such interventions can promote economic and social wellbeing, especially in rural communities whose identities and livelihoods are reliant on ecosystem services that require time spent outdoors. As such, additional research should explore the efficacy of protective actions and interventions on both physical health and wellbeing in the context of wildfire smoke air quality events.

In addition to providing community members with useful information to protect their health, CAMNs can enhance wildfire smoke exposure models and improve the understanding of the health and wellbeing impacts of wildfire smoke exposure [9]. A lack of standing infrastructure, research protocols, and data collection tools has been described as a key challenge for conducting timely health research in the aftermath of a disaster [27]. CAMNs may be an important tool in exposure assessment during wildfires and other disasters and hold promise for enhancing our understanding of disaster-related health impacts. In the context of a wildfire with significant air quality impacts, individual-level health impact information (e.g., biomarkers or self-reported health information collected through surveys) could be linked with exposure information from the nearest CAMN sensor. In order to rapidly and effectively collect information in the context of a real-world event with limited advance notice, associated protocols must be developed and validated, baseline information collected, and a provisional institutional review board secured.

### Limitations

The number of potential study participants was constrained by the inclusion criteria established in our purposive sampling approach: role as a clean air ambassador in Clean Air Methow’s Clean Air Ambassador Program. However, we were able to recruit 100% (*n* = 18) of the Clean Air Ambassadors to participate in our study. Prior research has demonstrated that thematic saturation—or when further data provides little or no additional information about research-relevant themes—can be achieved in as few as 12 interviews [28]. Further, while the use of a case study approach and qualitative research methods provides rich contextual data, it is limited in its generalizability. The motivations, uses, and challenges of citizen scientists volunteering to participate in CAMNs may be different in communities of different sizes and demographics. In addition, Clean Air Methow’s CAMN had only been existence for a short amount of time when the interviews were conducted, and interviews were conducted during a particularly bad wildfire season. The CAAs’ perspectives about and experiences with the CAMN may change over time, especially as they use the CAMN during other seasons. As we only interviewed CAAs who volunteered to host sensors, CAMN’s impacts and uses at the community-level may be different and should be the focus of future research.

## 5. Conclusions

The Clean Air Methow CAAs reported diverse motivations, data uses, and ideas for future project directions. Few challenges to participation or use of the network were described. The findings from this case study may inform other wildfire smoke-prone rural communities interested in establishing a citizen-engaged CAMN, including the recruitment of volunteer sensor hosts. Additional research is necessary to assess the efficacy of the protective actions taken in response to receiving air quality information from the CAMN on health and wellbeing, as well as to understand the broader community’s experience with and use of the CAMN.

## Figures and Tables

**Table 1:** Clean Air Ambassador demographic information.

	*n* *	%
**Number of People in Household**		
1	2	12
2	10	59
3	1	6
4	4	24
**Number of Children Under 18 in Household**		
0	11	65
1	3	18
2	3	18
**Marital Status**		
Single	1	6
Married	12	71
Separated or Divorced	2	12
Widowed	1	6
Partners	1	6
**Race**		
White	15	88
Black or African American	0	0
Asian	0	0
American Indian or Alaska Native	0	0
Prefer Not to Say	2	12
**Gender Identity**		
Female	12	71
Male	5	29
Non-binary	0	0
**Age**		
20–39	3	18
40–59	7	41
60–79	7	41
**Highest Educational Attainment**		
Some High School	0	0
High School or GED	0	0
Some College	2	12
Associates Degree	1	6
Bachelor’s Degree	10	59
Post-graduate Degree	4	24
**Employment Status**		
Employed full-time	6	35
Employed part-time	5	29
Seeking Opportunities	0	0
Retired	6	35

* Only 17 of 18 participants provided demographic information, as one participant hosted the sensor on behalf of their employer.

**Table 2:** Key themes in CAA-reported motivations for participation, data uses, future project ideas, and project ideas.

Domain	Themes Reported by CAAs
Motivations for Participation	Exposure reduction. Perceived personal and familial susceptibility to wildfire smoke. Interest in air quality or environmental science. Community benefits.
Data uses	Understand air quality conditions. Take protective actions. Engage in awareness building and community discussions around air quality.
Future project ideas	Expand use for other seasonal air quality issues. Use to promote awareness of other forms of pollution and inform or minimize pollution-generating activities. Use by different stakeholder groups. Educational opportunities in schools and the community. Increase number of air monitors within and outside of the community.
Project challenges	Accessibility limited to those with internet access. Concerns about sensor dependence on WiFi and electricity.
